# Finite element analysis on the human and guinea pig cochlear vibration patterns under bone conduction stimulations

**DOI:** 10.1038/s41598-024-76362-5

**Published:** 2024-10-27

**Authors:** Mingduo Zhao, Stefan Stenfelt

**Affiliations:** Department of Biomedical and Clinical Sciences, Linköping University, Stair D Level 11, 58185 Linköping, Sweden

**Keywords:** Bone conduction hearing, Vibration pattern of the cochlea, Finite element model, Human and guinea pig, Experimental models of disease, Computational biophysics, Computational biology and bioinformatics

## Abstract

To compare the vibrational patterns of human and guinea pig cochleae accurately, we developed and validated a novel finite element model of the guinea pig, leveraging it to analyze vibrational patterns in the cochlea. This approach is mirrored in our examination of the human cochlear model, providing granular insights into the nuances of human bone conduction hearing. The comparative analysis reveals that the guinea pig cochlea mirrors human cochlear vibrational patterns, thus serving as an efficient proxy for exploring human cochlear function. The human mastoid and the upper region of the guinea pig’s skull are recommended as the convenient and comparable sites for bone conduction stimulation. The cochlear vibration pattern encompasses a mix of rigid, rotational, and compressive motion. Significantly, the guinea pig model demonstrates robust agreement with existing experimental data and other studies, these findings are confirming the validity of the model. Our study delineates the distinct roles of the three vibration types across various frequency spectrums. At lower frequencies, rigid motion is the dominant mechanism, supplemented by rotational motion. However, at higher frequencies, the influence of rigid motion wanes, ceding prominence to rotational and compressive motions. This trend is consistently observed in both human and guinea pig models.

## Introduction

Bone conduction (BC) hearing, one of two primary auditory pathways, has been recognized for centuries and studied since the very beginning of modern auditory physiology^1,2^. In contrast to air conduction (AC) hearing, where sound waves travel through the air and are captured by the external ear (pinnae) and through the ear canal. Then it reaches the middle ear and causes the eardrum vibration, which is transmitted via the middle ear ossicular chain (malleus, incus, and stapes). Finally, it shakes the inner ear (cochlea) on the oval window, causing relative motion of the fluid and the basilar membrane to excite the hair cells and generate action potential, which goes to brain and becomes a hearing sensation. Bone conduction hearing is a type of hearing that occurs when sound waves are directly transmitted to the inner ear, mainly via the bones (primarily the skull bone), rather than through the air. It also finally results in vibrating the cochlea and exciting the hair cells, the hearing sensations are the same as normal AC hearing^3–8^.

To enhance auditory capabilities, BC hearing presents a beneficial approach, offering several advantages over AC hearing, such as circumventing the external and middle ear, directly stimulating the cochlea. Therefore, to deepen our comprehension of BC hearing, the vibration pattern evoked by the sound should be further investigated during bone conduction. However, there are some limitations and challenges in performing experiments on humans, for example, it is difficult to measure human cochlear vibration in living human and obtaining high-resolution frequency data. Therefore, this necessitates the use of animal models for reference. Based on our previous studies^7,8^, we selected the guinea pig, a species whose low-frequency hearing is similar to the human one^9^, as an animal reference, and primarily focused on the cochlear vibration patterns of the human and guinea pig, which play the most important role in the hearing sensations. To precisely describe the vibration patterns of the cochlea during bone conduction hearing, we used finite element model (FEM) of human and guinea pig to track and simulate every point on the model surface.

In pursuit of this understanding, we have developed a new finite element model (FEM) for the guinea pig cochlea. This model, aligned with existing experimental data, allows for precise depiction of cochlear vibrations during BC hearing. Previous studies and FEM simulations of human skulls have revealed several different skull vibration patterns that depend on frequency during BC stimulation^10^. At sub-400 Hz frequencies, the skull bone approximately follows the rigid body motion principle. When the frequency increases to 1 kHz, the skull vibration behaves like a mass- spring system, and the whole skull resonance appears. In this range, large parts of the skull move in phase, such as, the petrous part of the temporal bone in the cochlea. When the frequency comes between 1 and 2 kHz, the vibration transmits as a wave form in the cranial bone, and there are some differences between the cranial vault and the skull base. The velocity of the wave is almost constant at approximately 400 m/s in the skull, while in the cranial vault, it becomes a frequency dependent which is around 250 m/s at 2 kHz increasing to 300 m/s at 10 kHz^1,4,5,11^. Due to the varying features at different frequencies, we only possess general information within certain frequency ranges, which is based on data from human simulation computations or cadaver measurements. Therefore, the frequency resolution is low. Despite these insights, a comprehensive understanding of these vibration patterns across the frequency spectrum, especially in living humans and guinea pigs, remains elusive.

In this research, we aim to extract information on comparing human and guinea pig cochlear vibration patterns across a high-resolution frequency spectrum. By constructing an FEM and aligning our model with experimental results from guinea pigs and humans, if they do exhibit similar patterns, the guinea pig can serve as a good model for BC research. Such enhanced understanding and basic data promise significant contributions to BC hearing science, providing a detailed model for living human and guinea pig cochlear vibration patterns, and potentially paving the way for advancements in BC hearing aid technologies, ultimately offering benefits to those people with hearing impairments.

### The different factors of BC hearing

From early to recent literature, the perception of bone conduction sound has been categorized into various components, with classifications ranging from one to seven distinct contributors based on differing methodologies. However, our group’s research has led us to identify five primary anatomical components that play critical roles in BC sound perception: (1) sound pressure in the ear canal, (2) inertial forces of the middle ear ossicles, (3) inertial forces and fluid pressure of cochlear fluids, (4) alterations in the cochlear space, and (5) sound pressure transmission from the intracranial space^1,4,5,10,12^. Notably, contribution of the ear canal to BC hearing is approximately 10 dB lower than other components at frequencies below 2 kHz, aligns with them between 2 and 4 kHz, and diminishes significantly above 4 kHz, dropping to approximately 40 dB lower at 12.5 kHz^12,13^. The middle ear component is predominantly influenced by the inertial properties of the ossicles, particularly around the middle resonance frequencies of 1.5 to 3 kHz^5,14^. Cochlear fluid is the most important component to BC hearing perception, and is assumed to be incompressible. The fluid inlet and outlet between the oval and round windows of the cochlea, and the compliant structures and smaller inlets and outlets are collectively known as the third window^6^. Alterations in the geometry of the cochlea, including the cochlea compression and distortional component, are the result of wave vibration spreading in the skull bone encapsulating the cochlea and are more important at frequencies greater than 4 kHz^1,4,15^. Sound pressure transmission from the intracranial space, is not deemed the most critical component for BC sound perception based on clinical evidence^16^. Overall, BC hearing relies on bone vibrations at or close to the cochlea, suggesting that investigations of cochlear vibration patterns are important for obtaining a better understanding of BC hearing. This is our primary purpose of the current study.

The temporal bone, which is in proximity to the cochlea, exhibits notable similarities and differences between humans and guinea pigs. Both species possess five constituent parts of the temporal bone: the squama, petrous, mastoid, tympanic parts, and the styloid process in human, contrasted with the guinea pig’s a mastoid like process, a tympanic bulla, a tympanic ring, a petrosal segment, and a poorly developed squamosal portion. In addition, the topography of their cochlea is generally both snail-shaped. Despite these general similarities, several significant distinctions still exist. The most important factors affecting the vibration are the direction of the cochlear axis, the size and the number of snail turns in the cochlea: (1) the cochlear axis in guinea pigs is significantly less slanted than in humans; (2) the size of the cochlea in guinea pigs is much smaller than in humans (3) the number of snail turns is 2.5 in humans vs. 3.5 in guinea pigs. It is crucial to be aware of these differences and similarities when we consider the guinea pig as a model for human ear studies^17,18^. In fact, these differences have led to some intriguing discoveries finally.

### Vibration of the cochlea

In the realm of BC hearing, the predominant motion is that vibrations directly and mainly stimulate the cochlea through the skull bone^4,5^. For instance, the human cochlea can be conceptualized as a coiled spherical tube, approximately 10 mm in diameter. And it encompasses three parallel membranous, fluid-filled canals: the scala vestibuli (SV), scala media (SM), and scala tympani (ST). The SV and ST are filled with perilymphatic fluid, whereas the endolymphatic fluid resides within the SM, housing the organ of Corti (OoC), supported by the basilar membrane (BM) positioned between the SM and ST. However, the scala vestibuli and scala tympani differ in size, with the SV space being approximately 50% larger than the ST. Additionally, the stiffness of the oval window results in greater impedance compared to the round window. This differential in size and impedance leads to a fluid dynamic within the cochlea: under compression, excess fluid shifts from the SV to the ST and the round window, while during expansion, the fluid flow reverses^5,15^.

This vibrational process within the cochlea is multifaceted and encompasses various types of motion. Each type of motion plays a distinct role in the functioning of the cochlea. Therefore, we separate the motion patterns of the cochlea into three categories: rigid motion, rotational motion, and compressional motion^19,20^. Rigid motion is the entire cochlea moves in unison as a single entity, maintaining its shape, this motion is often referred to as common motion. Understanding rigid motion helps in understanding how the cochlea interacts with external forces. Rotational motion involves the cochlea twisting around a fixed point, altering its orientation. Studying rotational motion is essential for understanding the dynamics of how the cochlea responds to complex three-dimensional sound inputs. Compression motion is characterized by changes in the shape of the cochlear shell through compression or expansion along its axis. These distinct motion types exert varying influences on the fluid inside the cochlea based on the physical mechanism and consequently act on the OoC. This, in turn, modulates the hair cells’ response to sound, enabling them to attune themselves to different frequencies^19,20^. Classify in this way is helpful for our overall understanding the fundamental knowledge of hearing mechanics.

## Methods

**Fig. 1:**
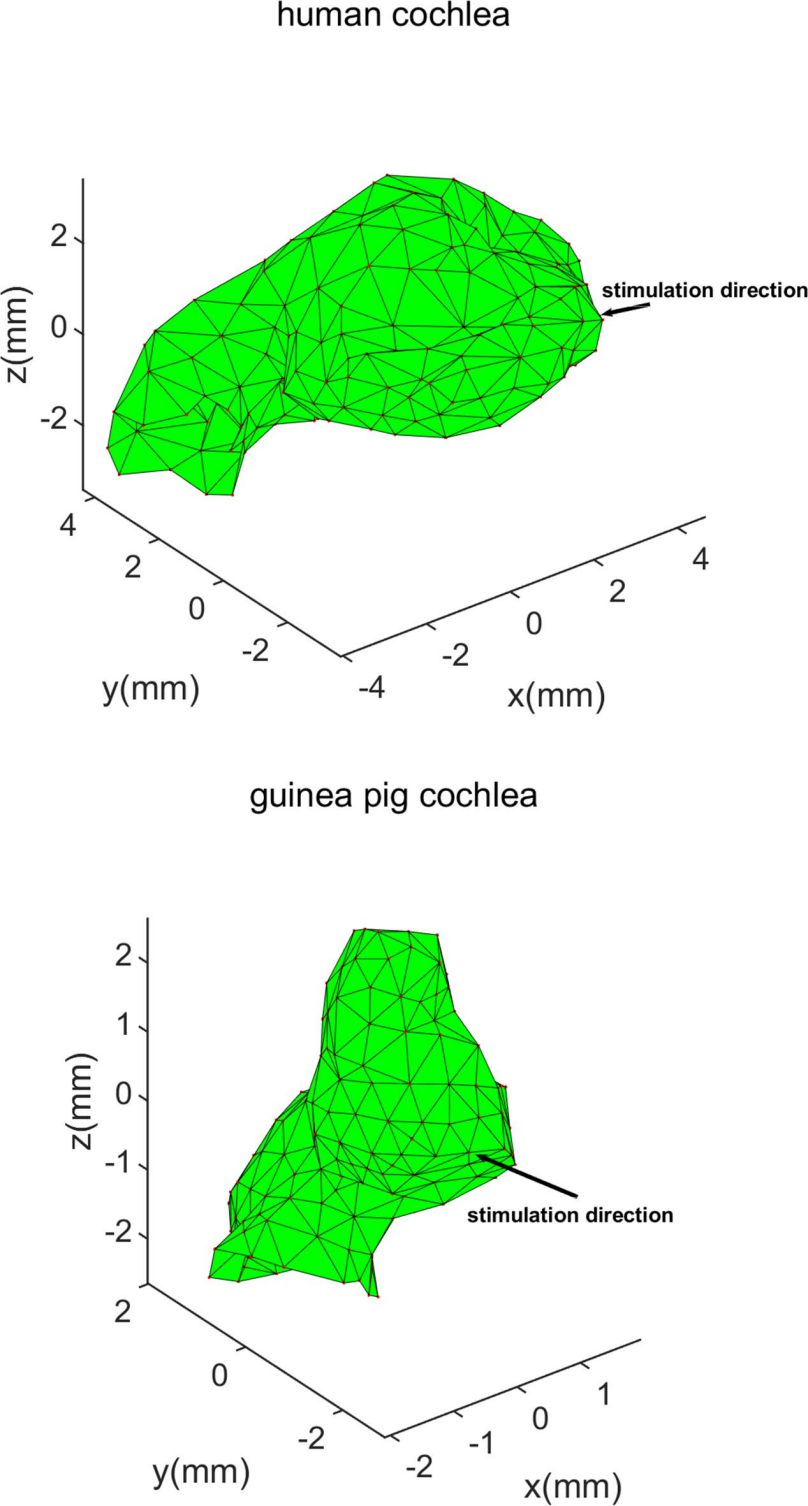
Human and Guinea pig cochlea models, and stimulation directions.

In our pursuit of elucidating the BC process, our laboratory previously developed the LiUHead model, a comprehensive whole human head finite element model (FEM), specifically tailored for investigations of BC sound transmission^21^. This sophisticated model integrates eight distinct domains: (1) soft tissue, (2) cartilage, (3) cortical bone, (4) soft bone, (5) cerebrospinal fluid (CSF), (6) brain and nervous tissue, (7) eyes, and (8) inner ears. By comprising 480,000 tetrahedral elements and 87,100 nodes, the LiUHead model adeptly simulates the vibrational dynamics of BC hearing^21^. In the present study, our focus was the cochlea, so we extracted the detailed cochlear section from the LiUHead model, which consisted of 247 nodes and 479 triangles. Refer to Fig. 1.

### Guinea pig finite element model

As the LiuHead, we used the same method to build a new similar model for the guinea pig. Through collaboration with Ghent University Centre for X-ray Tomography, CT images of an adult guinea pig head were obtained and used to reconstruct the geometry of the FE model. The original images had a voxel size of 0.045 × 0.045 × 0.045 mm. To get the intact geometry of the Guinea pig head, 2,940 images were used. The finite element model included six domains: (1) the brain, (2) eyeballs, (3) inner ears, (4) cartilages, (5) skull bone including teeth, and (6) soft tissues. The brain and part of the brainstem were modeled as brain tissue. The skull bone is modeled as a single layer structure comprising the skull and the teeth. The model contains the first vertebra. Auricular cartilage, as well as thyroid cartilage, is included around the ear canals. Due to the limitations of the scans and the complexity of the head anatomy, other tissues, such as skin, fat, muscle, blood, lymph, etc., are all modeled as soft tissue. This simplification also helps to reduce the demands on memory and time consumption for the computations. The final model consists of 203,000 four-node tetrahedron elements. The material properties of the model are different for every parts. The eyeballs and the inner ears are filled with liquids which have essentially the same properties as water and their material properties are modeled as water. The brain is modeled as an elastic material with low Young’s modulus. The skull bone including teeth is modeled as hard (cortical) bone with a Young’s modulus of 4GPa and a density of 2200 kg/m^3^. The cartilage in the model is assigned a density of 1000 kg/m^3^ and a Young’s modulus of 7.5 MPa. The soft tissues are modeled with a density of 900 kg/m^3^ and a Young’s modulus of 7 MPa. The boundaries are identified by the specific geometry image, and the FE model is freely movable at the outer boundary. The model was computed by a solver, using COMSOL Multiphysics (COMSOL Inc. Stockholm Sweden) with linear acoustics and solid mechanics modules. Preprocessing and meshing were conducted in HyperMesh (Altair Engineering, Troy, MI, USA).

The total force of the stimulation applied in both models was a dynamic force of 1 N. The stimulation point in the human coordinate system was on the right mastoid, and the accurate original unit vector was (0.9609, 0.2703, 0.06011) in the human coordinate system; In contrast, because the mastoid of the guinea pig is too small to fix the BC transducer, we found that the top of the guinea pig’s head is relatively flat and convenient for fixing the BC transducer. Thus, the stimulation point in the guinea pig model was on the top of the head, with the original unit vector being (0, 0, -1) in the guinea pig coordinate system. They are shown in Fig. 3. Stimulation was performed in the frequency domain from 0.1 to 20 kHz with a frequency resolution of 25 Hz up to 0.5 kHz, 50 Hz resolution between 0.5 and 1.0 kHz, and 100 Hz resolution above 1.0 kHz. After simulation and computation, we can obtain the velocity of every point in three dimensions as the amplitude and phase. From this guinea pig FEM, for this study, we extracted only the cochlear region, in which the border was between the bone and fluid, including 192 nodes and 370 triangles. See Fig. 1.

**Fig. 2:**
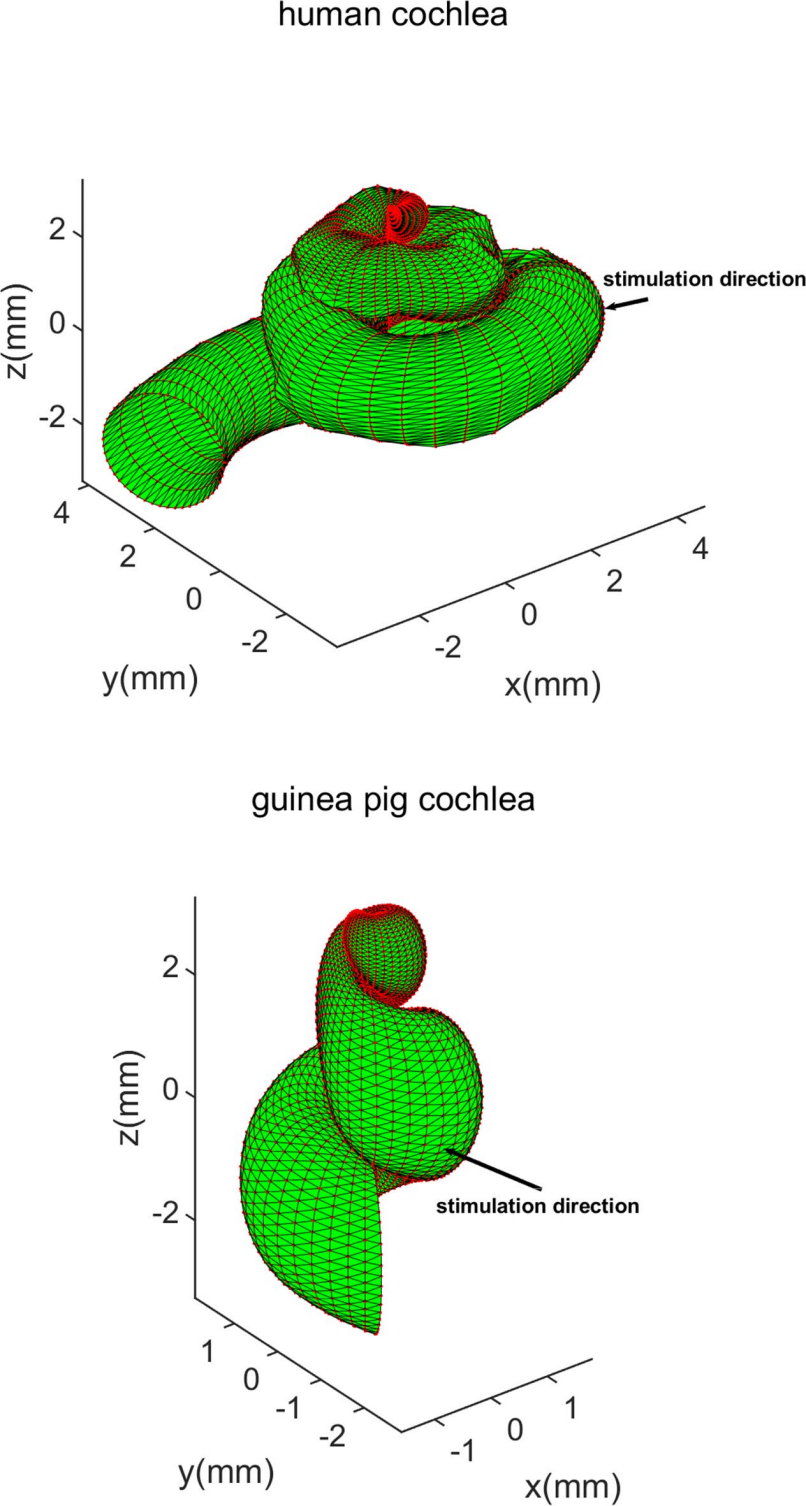
Parametric models of human and guinea pig cochlea, and stimulation directions.

While both human and guinea pig cochleae are snail-shaped, notable differences include the number of turns (2.5 in humans vs. 3.5 in guinea pigs) and size variations^18^. Furthermore, due to the low resolution of cryosectional images and CT scans, the models do not precisely represent the detailed geometry of the cochleae. To address this, we developed parametric models that fit the gross geometry of the original FEMs, enabling more accurate positioning and operation. However, these parametric models are not used for computation; the computations still rely on the original data points. Using parametric models, we can accurately identify the turns of the cochlea and determine the direction from the oval window to the round window. This information allows us to adjust and align the two cochlea models to the same position for comparison. The revised human cochlear model contains 5,251 nodes and 10,450 triangles, while the guinea pig model comprises 4,852 nodes and 9,653 triangles, as depicted in Fig. 2.

### Data analysis for 3D motion of cochlea

After the simulation experiments for the two FEMs, we acquired the velocity of every point in three dimensions, which included the amplitude and phase of the vibration velocities at all the model points for both the human and guinea pig. To better understand the complex mechanics of the cochlea and more effectively assess the different vibration, the cochlear vibration can be divided into 3 components: linear common motion (**v**_**o**_), rotational motion (**ω**_**o**_), compression differential motion (${\text{C}}_{\text{p}}$). Each component represents a distinct aspect of the motion that occurs in response to sound stimulation. Common motion refers to the synchronous movement of the entire cochlea, where the entire structure moves as a rigid body in response to sound vibration. The rotational motion refers to twisting movement of the cochlea around a pivot point, which is set at the middle of the cochlea’s central axis. Compression differential motion refers to the difference in motion amplitudes of the cochlear bone shell relative to the central axis, which can describe the compression and expansion of the cochlear shell.

We can use the following relationship equation to describe the velocity at any point, *p*:

1 $${\mathbf{v}}_{\mathbf{p}}={\mathbf{v}}_{\mathbf{o}}+{\boldsymbol{\upomega }}_{\mathbf{o}}\times {\mathbf{r}}_{\mathbf{op}}+{\mathbf{r}}_{\mathbf{op}}\cdot {\text{C}}_{\text{p}}$$

where **v**_**p**_ is the three-component velocity (*v*_*px*_, *v*_*py*_, *v*_*pz*_) at position p, **v**_**o**_ is the three-component translational velocity (v_ox_, v_oy_, v_oz_) at position O, **ω**_**o**_ is the three-component angular velocity (*ω*_*ox*_, *ω*_*oy*_, *ω*_*oz*_) about position O, and **r**_**op**_ is the vector between positions O and *p*. Therefore, we can transform the vector equation to the following equation for each component:

2 $$\begin{aligned}&{v}_{px}\left(x,y,z\right)={v}_{ox}+{\omega }_{oy}\cdot z-{\omega }_{oz}\cdot y+{x\cdot C}_{px}\\ &{v}_{py}\left(x,y,z\right)={v}_{oy}+{\omega }_{oz}\cdot x-{\omega }_{ox}\cdot z+y\cdot {C}_{py}\\ & {v}_{pz}\left(x,y,z\right)={v}_{oz}+{\omega }_{ox}\cdot y-{\omega }_{oy}\cdot x+z\cdot {C}_{pz}\end{aligned}$$

where *x*,* y* and *z* are the coordinates of different positions *p*, Eq. (2) can be written in matrix form to calculate as

3 $$\left[ {\begin{array}{*{20}c} {v_{{1x}} } \\ {v_{{1y}} } \\ {v_{{1z}} } \\ \cdot \\ \cdot \\ \cdot \\ {v_{{nz}} } \\ \end{array} } \right] = \left[ {\begin{array}{*{20}c} 1 & 0 & 0 & 0 & {z_{1} } & { - y_{1} } & {x_{1} } & 0 & 0 \\ 0 & 1 & 0 & { - z_{1} } & 0 & {x_{1} } & 0 & {y_{1} } & 0 \\ 0 & 0 & 1 & {y_{1} } & { - x_{1} } & 0 & 0 & 0 & {z_{1} } \\ \cdot & \cdot & \cdot & \cdot & \cdot & \cdot & \cdot & \cdot & \cdot \\ \cdot & \cdot & \cdot & \cdot & \cdot & \cdot & \cdot & \cdot & \cdot \\ \cdot & \cdot & \cdot & \cdot & \cdot & \cdot & \cdot & \cdot & \cdot \\ 0 & 0 & 1 & {y_{n} } & { - x_{n} } & 0 & 0 & 0 & {z_{n} } \\ \end{array} } \right]\left[ {\begin{array}{*{20}c} {v_{{ox}} } \\ {v_{{oy}} } \\ {v_{{oz}} } \\ {\omega _{{ox}} } \\ {\omega _{{oy}} } \\ {\omega _{{oz}} } \\ {C_{{px}} } \\ {C_{{py}} } \\ {C_{{pz}} } \\ \end{array} } \right]$$

or

4 $${\mathbf{V}}={\mathbf{B}}\cdot {\mathbf{A}}$$

According to the minimum square principle and system identification^14,22^, we can estimate each component in the following way:

5 $$\left[\begin{array}{c}{\widehat{v}}_{ox}\\ {\widehat{v}}_{oy}\\ {\widehat{v}}_{oz}\\ {\widehat{\omega }}_{ox}\\ {\widehat{\omega }}_{oy}\\ {\widehat{\omega }}_{oz}\\ {\widehat{C}}_{px}\\ {\widehat{C}}_{py}\\ {\widehat{C}}_{pz}\end{array}\right]={\left({\mathbf{B}}^{\mathbf{T}}\mathbf{B}\right)}^{-1}{\mathbf{B}}^{\mathbf{T}}\mathbf{V}$$

where ^–1^ indicates inversion of the matrix and T is the transpose of the matrix. These nine estimated parameters can then be used to calculate any point motion of the cochlea according to Eq. (2). The resulting velocity estimate at a position (x, y, z) on the cochlea becomes:

6 $$\begin{aligned} &{\widehat{v}}_{px}\left(x,y,z\right)={\widehat{v}}_{ox}+{\widehat{\omega }}_{oy}\cdot z-{\widehat{\omega }}_{oz}\cdot y+{x\cdot \widehat{C}}_{px}\\ & {\widehat{v}}_{py}\left(x,y,z\right)={\widehat{v}}_{oy}+{\widehat{\omega }}_{oz}\cdot x-{\widehat{\omega }}_{ox}\cdot z+y\cdot {\widehat{C}}_{py}\\&{\widehat{v}}_{pz}\left(x,y,z\right)={\widehat{v}}_{oz}+{\widehat{\omega }}_{ox}\cdot y-{\widehat{\omega }}_{oy}\cdot x+z\cdot {\widehat{C}}_{pz}\end{aligned}$$

**Fig. 3:**
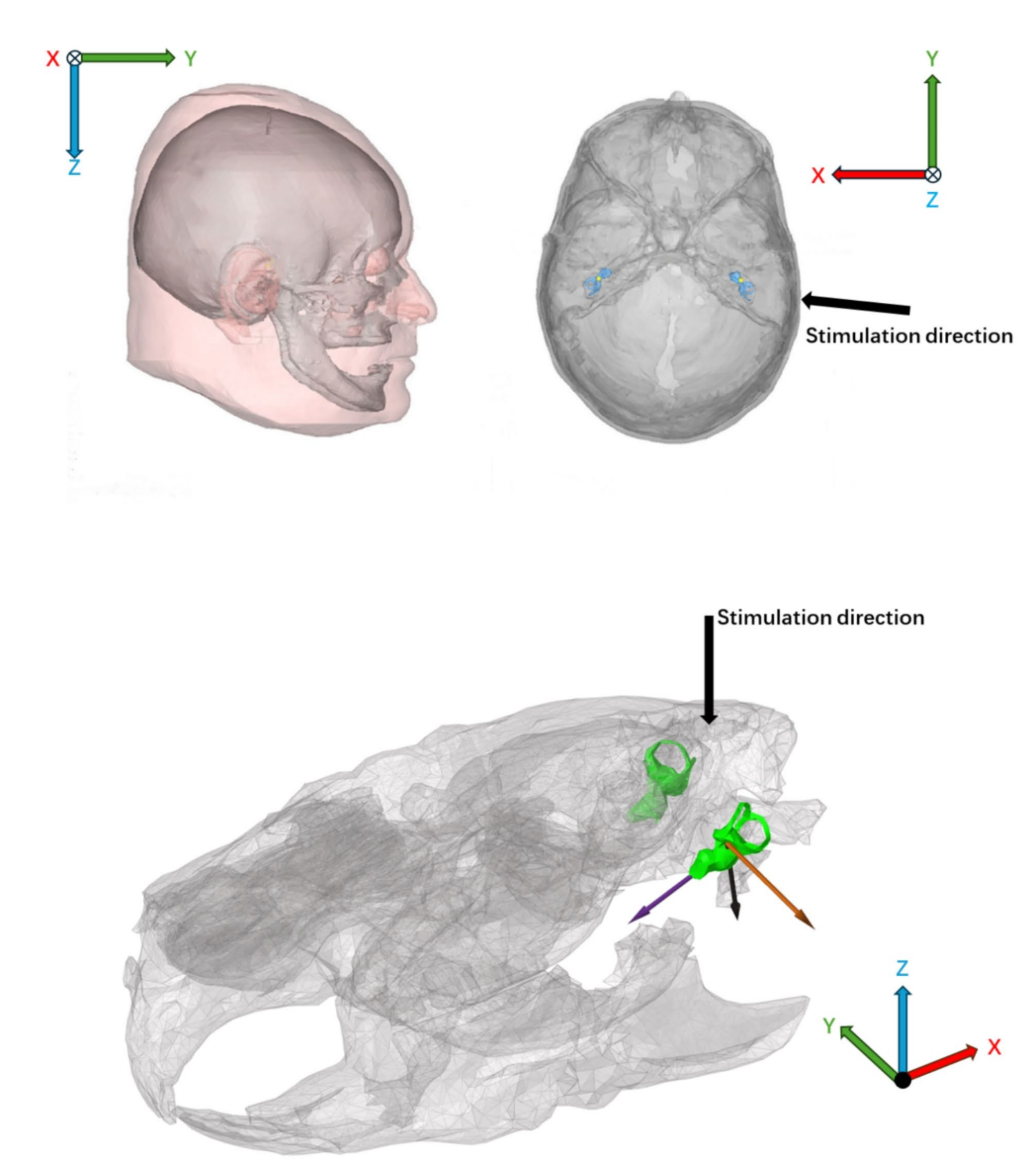
Human and guinea pig local coordinate systems, and stimulation directions. Models were created in HyperMesh 14.0 https://altair.com/hypermesh.

According to these math methods and all the points’ simulation result data (247 points for the human and 192 points for the guinea pig), we used MATLAB to compute the estimated values for the 3D translational, rotational, and compressional components. Because there were some differences between the two models, including the local coordinate system (Fig. 3), the direction of the cochlear center axis, the spiral direction of the cochlea, the direction from oval window to round window and the stimulation direction. We transformed the human coordinate system to the guinea pig local coordinate system and position for both cochleae in the same direction. The results for both cochleae are presented in an xyz coordinate system: the x-axis for each cochlea is along the direction from oval window to round window, the z-axis is along their center axes which is from the bottom center to the apex of the cochlea, and the origin point, O (0, 0, 0), is set at the middle of the cochlea’s central axis, the y-axis is perpendicular to the xz plane at point O (Fig. 1). After these steps, we found that the angle between the two stimulation directions was 52.78° and they were almost both in the xy plane, the intersection angle between the xy plane and stimulation for the human was 4.6°, while that for the guinea pig was − 2.8°, almost perpendicular to the z direction. The angle of 52.78° comes from anatomical differences between human and animal cochleae.

For compression component analysis, some radius data of the human and guinea pig cochleae are required to estimate compression. Since ${\text{C}}_{\text{p}}$ does not have a complete unit, it needs to be multiplied by the radius of the point to obtain the unit of mm/s. We use the maximum radius of the cochlea to represent the compression vibration as it is the most prominent point. According to our model and the findings of several previous studies, the maximum radii of the human cochlea are approximately 5 mm^10^ and 2 mm in the guinea pig^23^.

## Results and discussion

**Fig. 4:**
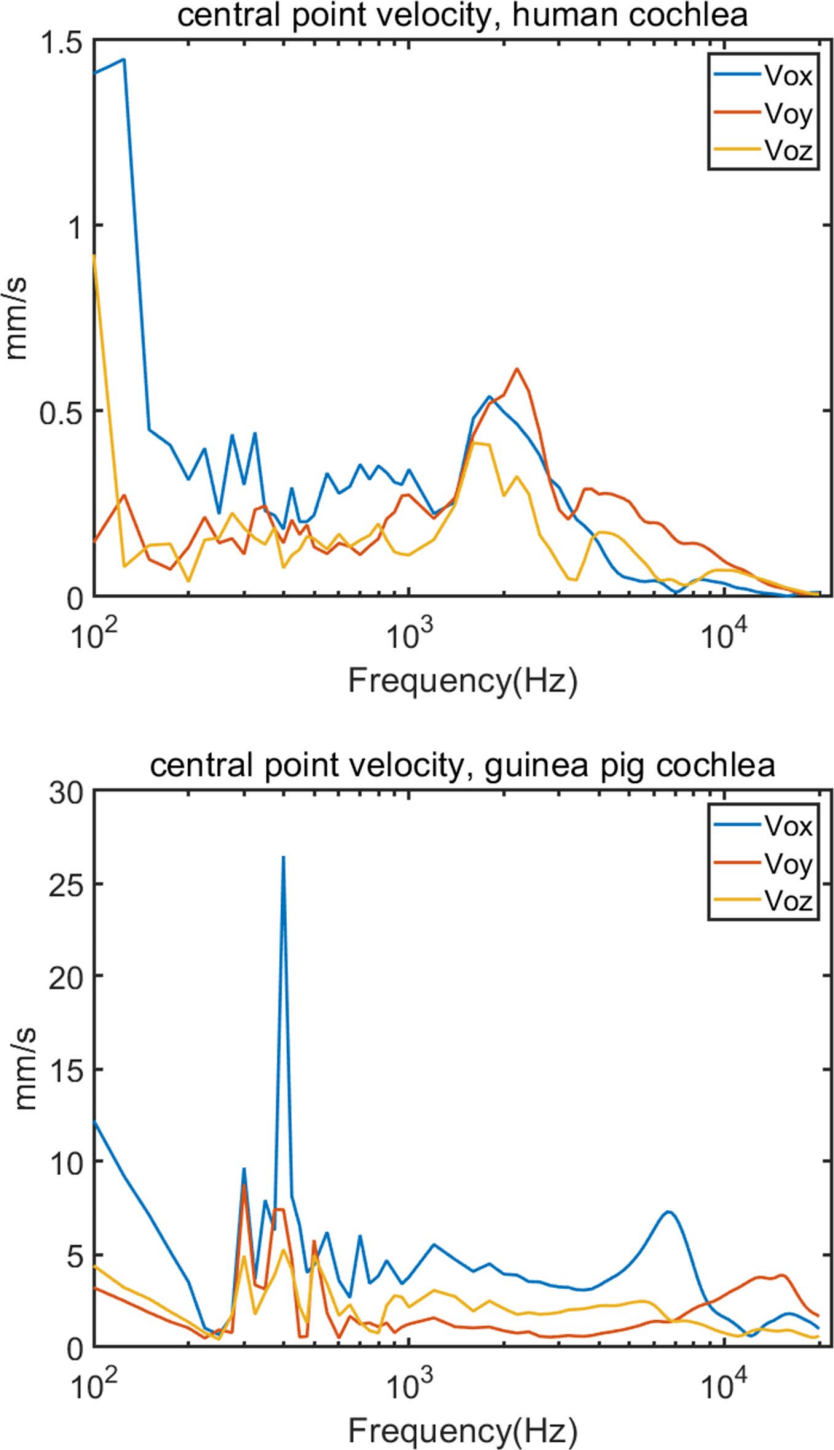
Central point velocity of human and guinea pig cochlea vibration.

**Fig. 5:**
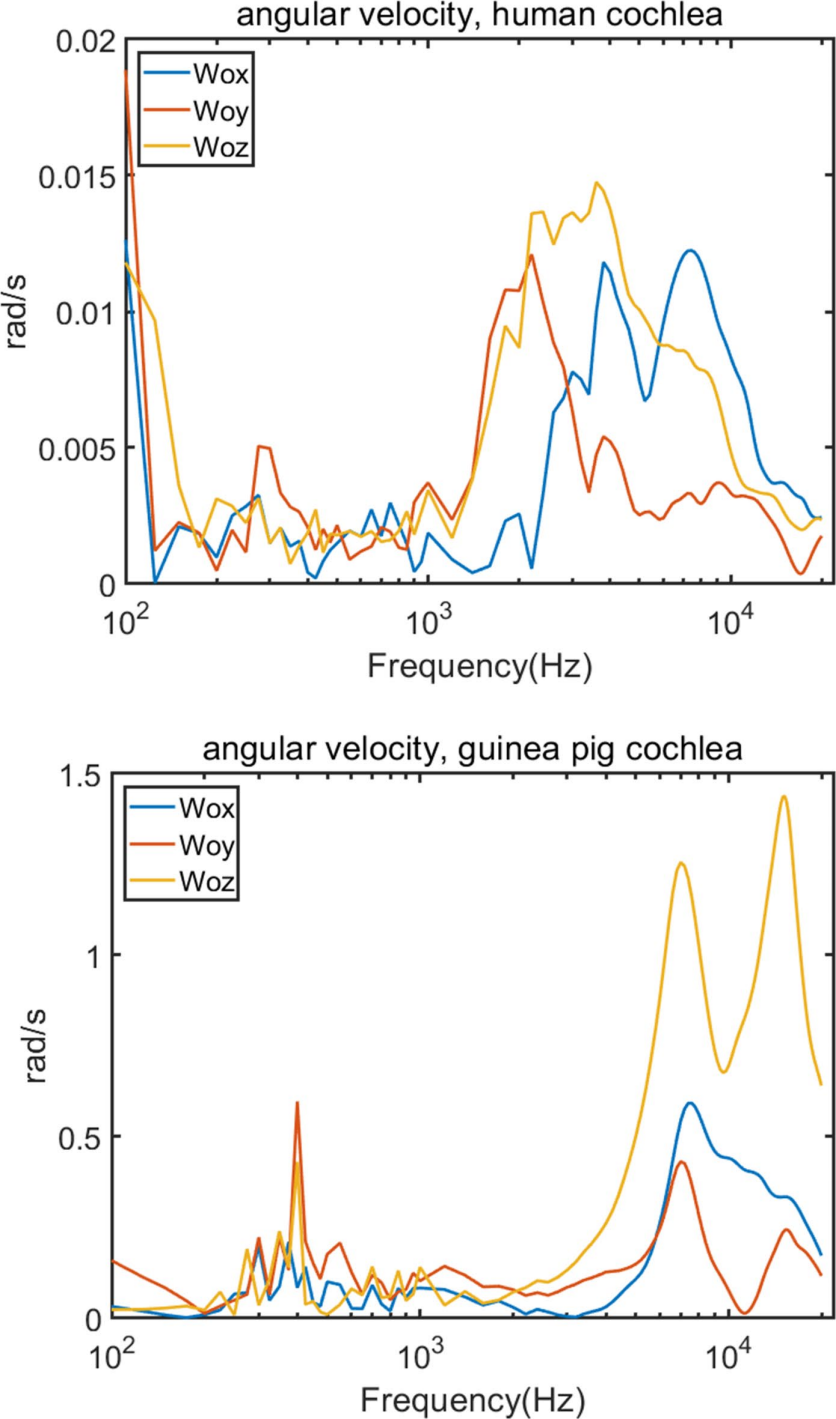
Angular velocity of human and guinea pig cochlea vibration.

**Fig. 6:**
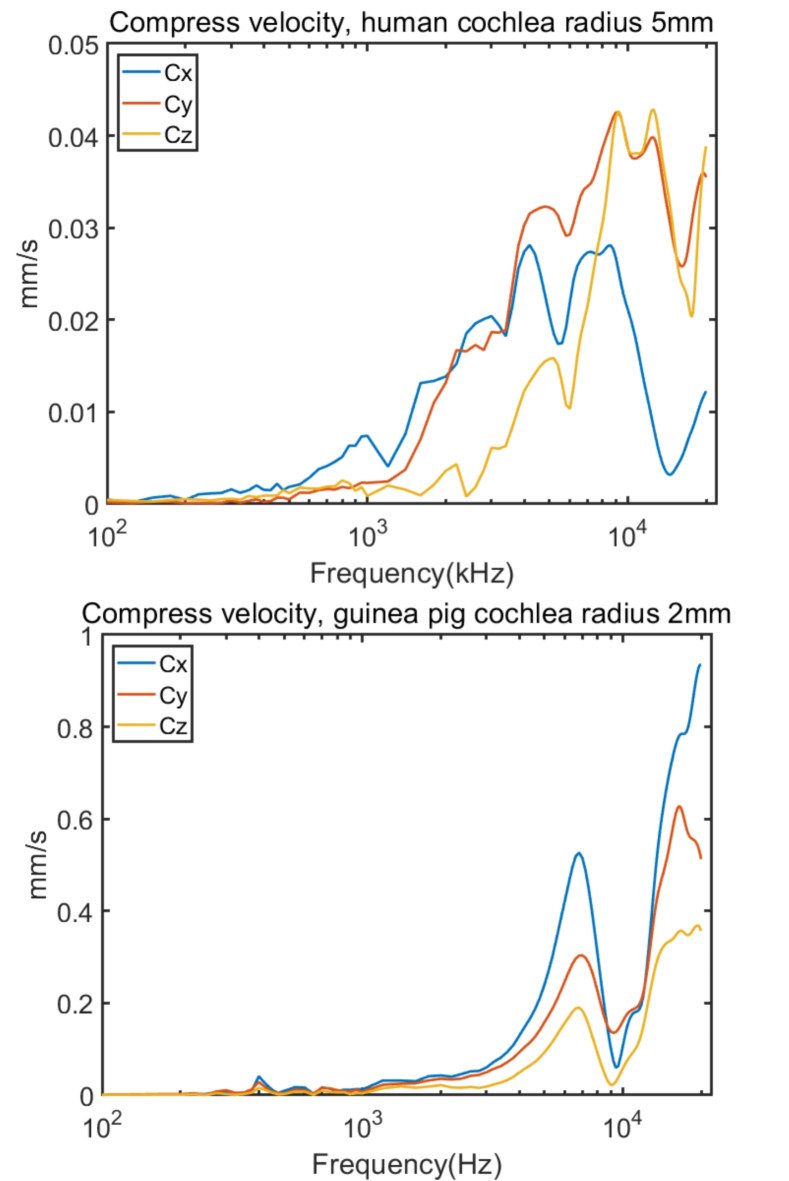
Compress velocity of human and guinea pig cochlea vibration.

Following the outlined methodology, we computed the motion raw data and estimated the components **v**_**o**_, **ω**_**o**_, and ${\text{C}}_{\text{p}}$ for both human and guinea pig cochleae. To facilitate a comparative analysis, these results are depicted in Figs. 4 and 5, and 6.

Figure 4 illustrates the central point velocities (**v**_**o**_) between human and guinea pig cochleae. Notably, the human cochlear velocity is significantly lower than that of the guinea pig in general, which is attributable to the greater mass of the human head relative to that of the guinea pig, despite identical 1 N force application in both cases. Furthermore, the figure elucidates the relative velocity distribution across the x, y, and z directions. At lower frequencies, the x directional velocity predominates in both models, with y and z directional velocities exhibiting comparable levels. However, at higher frequencies (approximately 3 kHz to 5 kHz for humans and 6 kHz to 12 kHz for guinea pigs), the x-directional velocity decreases sharply, supplanted by the y-directional velocity. Additionally, there was a notable peak around 400 Hz in the x directional velocity for the guinea pig model.

Figure 5 compares the angular velocities (**ω**_**o**_) between the two species, with human values again being lower for the same reason as the velocities of the central point. At the first data point, 100 Hz, there is a scientifically high peak. The reason may be that, when the simulation begins, the system transitions from a state of an initial condition to a state of vibration. This sudden transition can cause transient effects, where the system has not yet reached a steady-state vibrational mode. The first data point might capture these initial transient behaviors, which can be unrepresentative or abnormal for the first data point. Therefore, we will not consider the data result at 100 Hz. After this, at low frequencies, both human and guinea pig data show a peak around 300–400 Hz. After this peak, the values decrease with some fluctuations. However, at high frequencies, both velocities increase significantly and reach a second peak. Moreover, at higher frequencies, the wavelength of the sound wave is shorter, because the shell of the cochlea is a stiff structure, moreover, the shape of the cochlea is a cone which the distance between the outer wall of the cochlea decreases as the cochlea moves toward the apex. This makes the cochlea vibrate more quickly to respond to the sound, especially as the frequency increases. This makes the apex of the cochlea vibrate more quickly than the bottom, resulting in the cochlea twisting and the angular velocity growing up as the frequency increases. These findings are in accordance with our angular velocity simulation results. Additionally, at high frequencies in guinea pig, our simulation shows there are two peaks approximately 7 and 16 kHz. These peaks were found in our previous studies^7,8^, in which we measured the velocity of guinea pig cochlear vibration and found two distinct peaks at approximately 8 kHz and 16 kHz, respectively, accompanied by a notable trough at approximately 10 kHz. Because in the previous experiment, we only measure 2, 4, 8, 12, 16, and 20 kHz frequencies, they are not continuous and make the comparisons slightly different^7,8^. However, the trend and amplitude change in the vibration velocity are in accordance with our simulation. These consistent findings show that angular velocity contributes to both low and high frequencies in BC hearing and plays a more important role in high frequencies. Moreover, at low frequencies, the angular velocity in the y direction is the highest, while that in z direction is the highest at high frequencies. These situations occur in both the human and guinea pig.

In Fig. 6, the compression velocities (${\text{C}}_{\text{p}}$) of the human cochlea are again lower than those of the guinea pig for the same reason as the velocities of the central point. When plotted on a logarithmic scale, both species exhibit a similar trend of increasing compression velocities with frequency, with some fluctuations, among the three directions, they follow nearly the same trend. A further comparison of the resultant velocity of compression between the human and guinea pig yielded a correlation coefficient of 0.89. This correlation coefficient is high, indicating that the human and guinea pig have a similar trend in compression velocities according to frequency. Additionally, in detail, we also found a small peak at approximately 400 Hz in the guinea pig results. The reason we observed a peak at approximately 400 Hz in all 3 types of responses was that the guinea pig responded to this frequency as an integral. This is probably a result of the vibration of the entire guinea pig’s head, likely resulting from a rotational response. At high frequencies, from 2 kHz in the human and 3 kHz in the guinea pig also exhibited a similar trend that they both drastically increased. Moreover, there are two noteworthy peaks at approximately 7 and 16 kHz and one trough at approximately 10 kHz. These results are in accordance with the experimental results obtained for angular velocities and indicate that the compression velocity also contributes slightly to approximately 400 Hz and more to high frequencies. However, in the human cochlea, the y and z directions are the higher than the x direction in high frequencies, while the x direction is the highest in the guinea pig. And both have a valley for 3 directions at high frequencies.

**Fig. 7:**
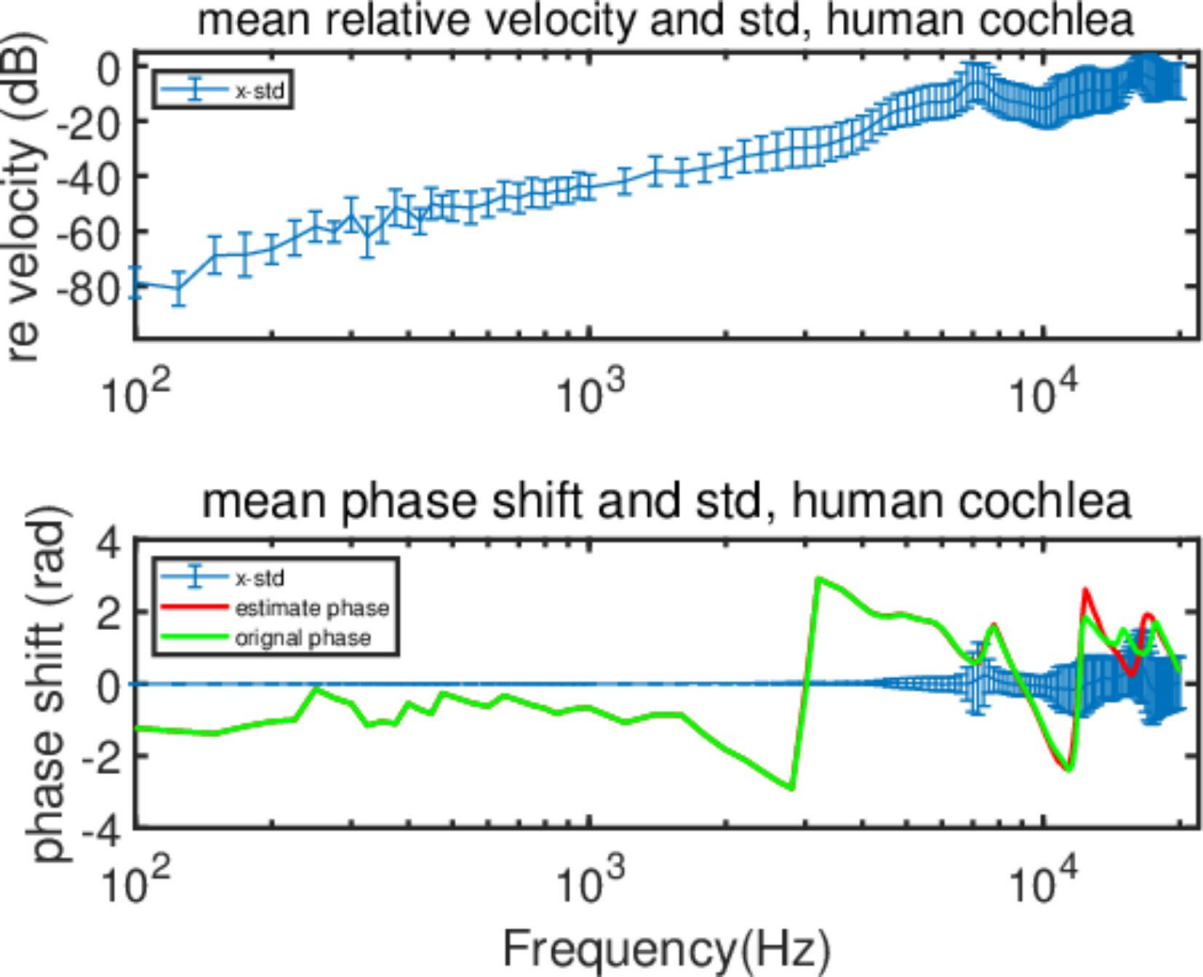
Mean relative velocity and phase shift with std of human cochlea vibration.

**Fig. 8:**
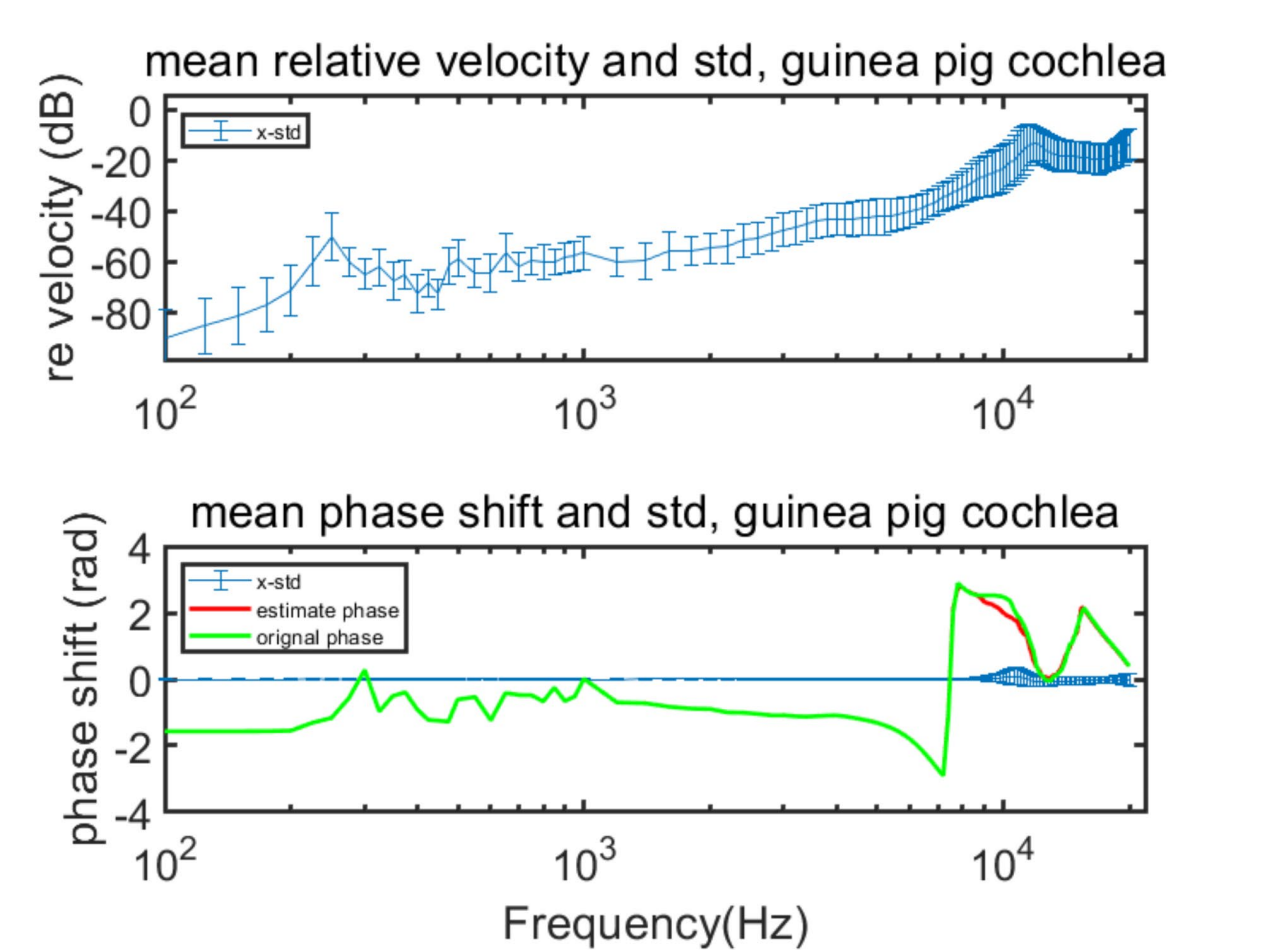
Mean relative velocity and phase shift with std of guinea pig cochlea vibration.

After obtaining **v**_**o**_, **ω**_**o**_, and ${\text{C}}_{\text{p}}$ results for both species, we applied Eq. (6) to estimate the motion of the cochlea. By comparing the mean amplitude and phase values between the estimated and original data using the Pearson Correlation Coefficient, we observed near-perfect correlations, all greater than 0.9999, indicating the accuracy of our simulations. Furthermore, the relative error velocities, presented in dB scale (20*lg((V_estimated_-V_original_)/V_original_)), generally increase from approximately − 80 dB to -20 dB with frequency, such as x direction of the human and guinea pig shown in Figs. 7 and 8. These findings confirm that the amplitudes of the estimated and original data are consistent, especially at lower frequencies. Phase shifts (PH_estimated_-PH_original_) across frequencies and differences between estimated and original phase are shown in Figs. 7 and 8. These differences are minimal and the Pearson Correlation Coefficient between the estimated and original phases are all greater than 0.9758. These high correlation coefficients further validate the alignment between the estimated results and original data, barring minor fluctuations at very high frequencies.

**Fig. 9:**
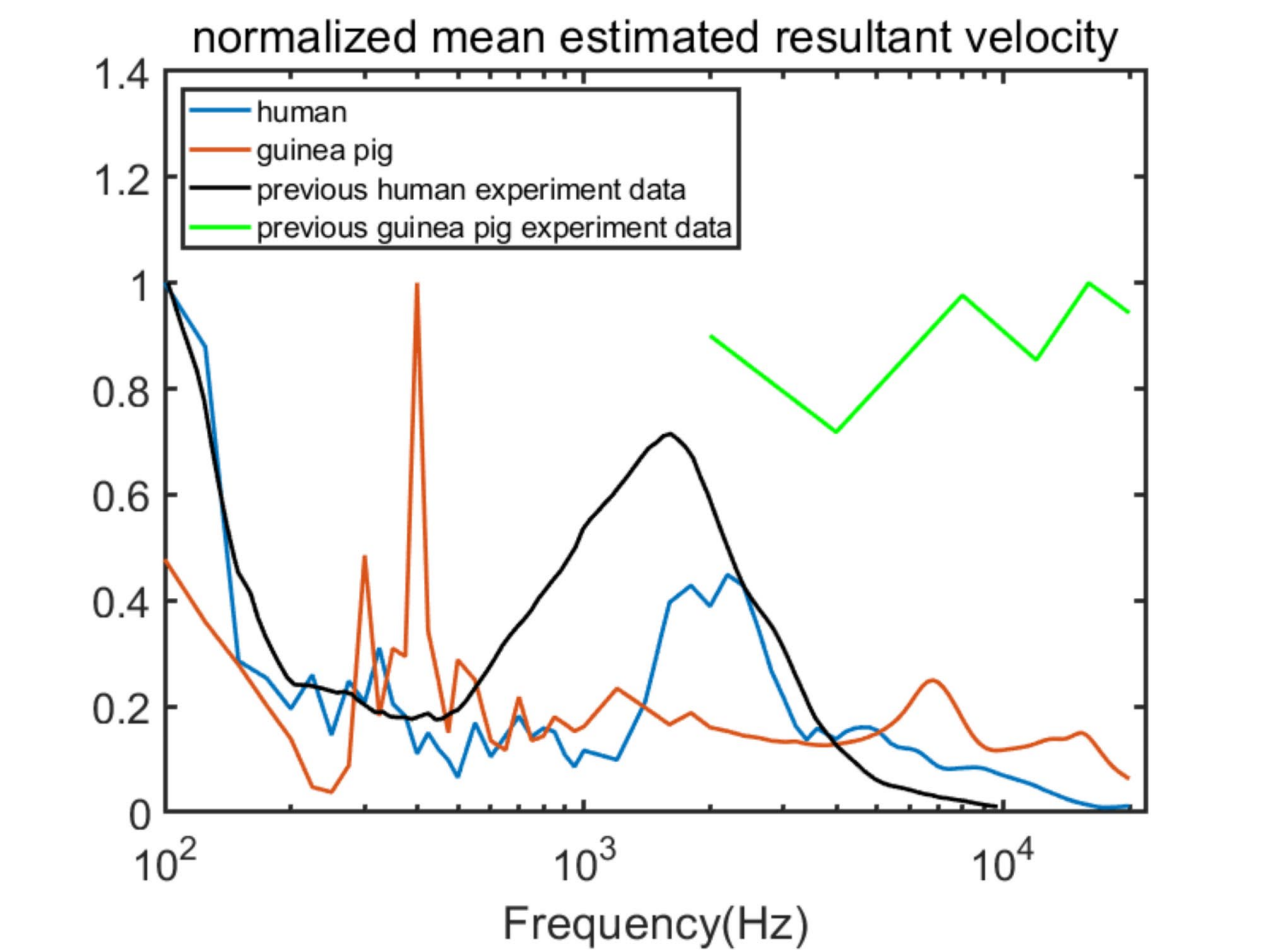
Normalized relative mean estimated resultant velocity between human and guinea pig.

Upon completing individual analyses, to intuitively contrast the resultant vibration patterns of human and guinea pig cochleae, we normalized their resultant velocities (${\mathbf{v}}_{\mathbf{p}}=\sqrt{{{\varvec{v}}_{\varvec{px}}^{2}}+{{\varvec{v}}_{\varvec{py}}^{2}}+{{\varvec{v}}_{\varvec{pz}}^{2}}}$) and previous guinea pig experimental data for comparison by ${\mathbf{v}}_{\mathbf{p}}/{\mathbf{v}}_{\mathbf{p}\mathbf{m}\mathbf{a}\mathbf{x}}$, as shown in Fig. 9. These findings are consistent with prior human and guinea pig experimental outcomes^7,8,19,21^. In previous studies^7,8^, the focus was on frequencies between 0.1 and 10 kHz for humans and between 2 and 20 kHz for guinea pigs. In our simulations, human cochlear responses exhibit a decrease starting from 0.1 kHz, reaching a trough at approximately 0.4 kHz, followed by an increase to a peak near 2 kHz, before tapering off at higher frequencies. This pattern aligns with earlier findings^19,21^. In guinea pigs, from 2 kHz, both our simulation and previous experimental data decrease until 4 kHz, and then increase to a peak at approximately 7 kHz; after this, they decrease to a trough at approximately 10 kHz, climb again and reach the second peak at approximately 16 kHz, and finally decline again. Because in the previous experiment^7,8^, we only measured 2, 4, 8, 12, 16, and 20 kHz frequencies, there is no data available for 100–400 Hz, where the highest peaks appear in the other three datasets. I could only select the highest value within the measured frequencies and then normalize the velocity by ${\mathbf{v}}_{\mathbf{p}}/{\mathbf{v}}_{\mathbf{p}\mathbf{m}\mathbf{a}\mathbf{x}}$​​. This makes the previous guinea pig experiment data appear much higher than the others. Additionally, since the experimental data is discontinuous, the curve is not smooth enough. These all make the comparisons slightly different. However, the trend and amplitude change are in accordance with the guinea pig FEM simulation result. Our simulations of the cochlear resultant velocity for the human and guinea pig match well with the previous research, they both have a similar trend and amplitude changes according to frequencies, and at certain frequencies, they have similar peaks and troughs. These findings further prove the accuracy of our guinea pig FEM and our method for estimating the cochlear vibration. They can accurately describe and anticipate the details of cochlear motion in both humans and guinea pigs.

By comparing the resultant velocities between the human and guinea pig, this analysis revealed commonalities and differences in their cochlear responses. Both species demonstrate fluctuating vibration velocities across frequencies, indicating a complex cochlear response. Generally, vibration velocities fluctuate with increasing frequency in both species. However, the specific frequencies of the peaks and troughs differ. For example, humans exhibit a pronounced peak at approximately 2 kHz, while guinea pigs exhibit significant peaks at approximately 300–400 Hz, and smaller ones at approximately 7 and 16 kHz. Above 5 kHz, guinea pigs consistently exhibit higher vibration velocities than humans. These distinctions may be the pure mechanics resulting from the differences in geometry.

**Fig. 10:**
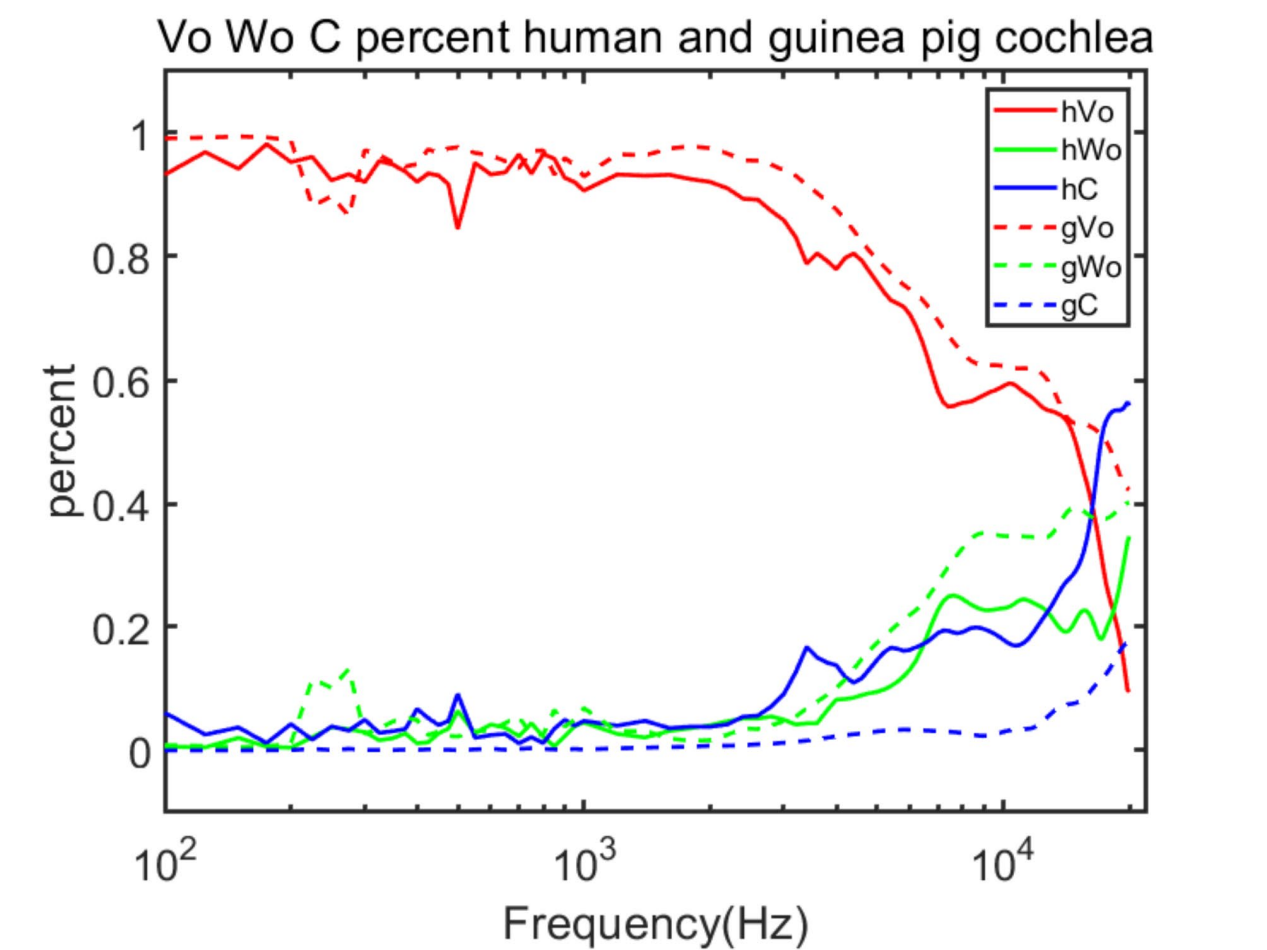
${\mathbf{v}}_{\mathbf{o}}$, ${{\varvec{\upomega}}}_{\mathbf{o}}$, ${\text{C}}_{\text{p}}$ percent in human and guinea pig cochlea vibration.

We further investigated the contributions of **v**_**o**_, **ω**_**o**_, and ${\text{C}}_{\text{p}}$ to the resultant velocities in humans and guinea pigs, as illustrated in Fig. 10. Based on this figure, particularly at frequencies below 7 kHz in humans and 8 kHz in guinea pigs, the **v**_**o**_ component plays a predominant role in both human and guinea pig models. Beyond these frequencies, the influence of **v**_**o**_ diminishes. In humans, its effect nearly vanishes at certain high frequencies, whereas in guinea pigs, it retains some presence. The difference likely stems from the disparity in cochlear mass between the species. When the mass is heavier, by the same force, it is difficult to vibrate quickly as an integral, especially it cannot respond well to high frequencies. That is why at the very high frequencies, the **v**_**o**_ of guinea pig cochlea still retains some presence. For the same species, the reason is similar, with the same mass, as the frequency increases, the common vibration (**v**_**o**_) becomes weaker. This makes ${\mathbf{v}}_{\mathbf{o}}$ take more proportion at low frequencies for both the human and guinea pig. For **ω**_**o**_, in both humans and guinea pigs, below 4 kHz and 3 kHz respectively, the role of the **ω**_**o**_ component is minimal. However, beyond these frequencies, its significance increases rapidly, indicating a more prominent role of rotational motion at relatively higher frequencies. Notably, in guinea pigs, an exceptional peak at approximately 400 Hz may stem from a rotational component **ω**_**o**_ of the entire guinea pig head. Additionally, at higher frequencies, particularly at approximately 8 and 16 kHz, we observe two minor peaks. These findings align with our previous guinea pig experimental results^7,8^, and further emphasize the increased contribution of rotational motion at these higher frequencies. This is quantitatively supported by the dramatic increase in the contribution percentage of **ω**_**o**_, which escalates from 3.8 to 34.7% in humans and from 1.7 to 40.1% in guinea pigs, beyond 2 kHz. For ${\text{C}}_{\text{p}}$, the situation is similar to that of **ω**_**o**_, before 2 kHz, its contribution is negligible in both humans and guinea pigs, and almost nonexistent. However, above 10 kHz, there is a dramatic increase. These findings highlight the significant role of ${\text{C}}_{\text{p}}$ at higher frequencies. Specifically, after 2 kHz, the contribution of ${\text{C}}_{\text{p}}$ increases from 3.8 to 55.8% in humans and from 0.8 to 17.7% in guinea pigs. Considering these velocity characteristics and the contributions of **ω**_**o**_ and ${\text{C}}_{\text{p}}$, **ω**_**o**_ and ${\text{C}}_{\text{p}}$ predominantly influence, and may even determine, all the observed peaks in the experimental data. Finally, to compare the contributions between the human and guinea pig, we calculated the Pearson Correlation Coefficient for ${\mathbf{v}}_{\mathbf{o}}$, **ω**_**o**_, and ${\text{C}}_{\text{p}}$ between the two species. The corresponding results are 0.9607, 0.9510 and 0.9779, respectively. These high Pearson Correlation Coefficients show that the ${\mathbf{v}}_{\mathbf{o}}$, **ω**_**o**_, and ${\text{C}}_{\text{p}}$ have similar contributions to the resultant velocities between humans and guinea pigs.

In summary, our simulations revealed both similarities and differences in the cochlear vibration patterns of humans and guinea pigs.

For the common points, for the relative velocities of the central point, the velocity in the x direction is the highest, that in the z direction is in the middle and that in the y direction is the lowest for most frequencies. At high frequencies, the x and y directions exchange positions, the y direction increases to the highest position while the x direction decreases to the lowest position. These situations happen in both the human and guinea pig results. The common velocity component of both humans and guinea pigs constitutes a significant majority of the resultant velocities at low frequencies. However, its impact starts to diminish beyond the 3–4 kHz range. Similar trends are observed in angular velocities for both humans and guinea pigs. At low frequencies, both have peaks at approximately 300–400 Hz, and at high frequencies, both increase substantially and have their second peaks. The compression velocities also follow a similar trend in which the compression velocities increase with some fluctuations, especially over 2 kHz, they drastically increase considerably, and both exhibit a trough at approximately 10 kHz at high frequencies. These similar patterns make the guinea pig a good model for human hearing research.

For the different parts, although the total force of the stimulation in the two models is the same, 1 N, the place on which the transducer is applied is different. The difference in size and mass of the cochlea only affects the vibration results in terms of magnitude by the same force, but it does not impact the trends change and relative comparison. While the difference of the transducer location, in the human model, the stimulation is from the right mastoid, and it is almost in the x direction in the human model coordinate system, while the stimulation in the guinea pig model was applied on the top of the head. After transposing the human cochlear model to the guinea pig cochlear local coordinate system, and adjusting these two models, we found that the angle between the two stimulation directions was 52.78° and both were almost in the xy plane, perpendicular to the z direction, the central axis of the two cochleae, as shown in Fig. 1. Usually, the vibration velocity is the highest in the stimulation direction, but the computed results do not reveal that the z direction has much greater vibration velocity compared to the x and y directions. This discrepancy could be because the original stimulation was applied in the z direction within the guinea pig model’s coordinate system, not in the guinea pig cochlear local coordinate system. After transforming the data to the guinea pig cochlear local coordinate system, the stimulation direction no longer aligned with the z direction. Since cochlea is a snail-shaped which is a repetitive structure, it is almost round shape in the xy plane and the stimulations of the human and guinea pig are both applied in the xy plane, the radial direction in the round plane of the cochlea, this perhaps makes the two stimulations not too different for the cochlea. Based on these findings, we can recommend the chosen stimulation sites for bone conduction stimulation. These sites are convenient and comparable locations for experimental operations and ensure that the stimulation produces similar effects. Moreover, we also found peaks at approximately 300–400 Hz in the guinea pig central point velocity and compression velocity results. At high frequencies, these peaks and trends are in accordance with previous experimental result^7,8,24^. This finding again verified the accuracy of our guinea pig FEM.

## Conclusion

In conclusion, our study revealed that the human cochlea and guinea pig cochlea do exhibit similar vibration patterns during BC hearing, and the guinea pig can serve as a good model for human BC research. The guinea pig cochlea finite element model serves as an effective tool for advancing human cochlear research. This model meticulously and accurately delineates the vibrational patterns of the guinea pig cochlea, offering valuable insights for analogous human studies. Notably, the chosen stimulation sites, the mastoid in humans and the top of the head in guinea pigs, are recommended, they are convenient and comparable locations for bone conduction stimulation. Furthermore, we observe that, at lower frequencies, the common velocity is predominantly influential; oppositely both the angular velocity and compression velocity are more crucial in high frequency hearing. These trends and patterns are consistent across both the human and guinea pig. All these findings offer a robust framework for future research and potential advancements in hearing aid technologies which may ultimately offer substantial benefits to people with hearing impairments.

## Data Availability

The datasets generated during and/or analyzed during the current study are available from the corresponding author on reasonable request.
